# Exome Sequencing Identifies a Novel Sorting Nexin 14 Gene Mutation Causing Cerebellar Atrophy and Intellectual Disability

**DOI:** 10.1155/2018/6737938

**Published:** 2018-10-24

**Authors:** Nadia Al-Hashmi, Mohammed Mohammed, Salim Al-Kathir, Naeema Al-Yarubi, Patrick Scott

**Affiliations:** ^1^Child Health Department, Royal Hospital, Muscat, Oman; ^2^Molecular Genetics and Genomics Laboratory, Sultan Qaboos University Hospital, Muscat, Oman

## Abstract

The autosomal recessive cerebellar ataxias (ARCA) affect both the central and the peripheral nervous systems. They are also characterized by a relatively high level of genetic heterogeneity with well over 40 genes already implicated. The present study aimed to identify the gene mutation responsible for a complex phenotype comprising cerebellar ataxia and intellectual disability segregating in an Omani consanguineous family. Homozygosity-guided exome data analysis identified a novel frameshift mutation (c.2319_2322del) within the sorting nexin 14 gene (SNX14), which predicts complete absence of the SNX14 encoded protein. Segregation within the family of the sequence variation is consistent with its pathogenic role. Importantly, loss-of-function mutations in SNX14 have recently been described as a cause of a clinically distinguishable recessive syndrome consisting of cerebellar atrophy, ataxia, coarsened facial features, and intellectual disability. This study expands the genetic diversity of ataxia genes in the Omani population and have important implications for the clinical and molecular diagnosis of this condition in affected individuals.

## 1. Introduction

The ARCAs are a heterogeneous group of rare neurological disorders affecting both the central and the peripheral nervous systems [1, 2]. They are characterized by imbalance, poor coordination, and atrophy of the cerebellum. A new ARCA entity affecting the autophagy pathway has recently been described [3, 4]. This clinically distinguishable recessive syndrome consists of cerebellar atrophy, ataxia, coarsened facial features, and intellectual disability and is due to loss-of-function mutations in the sorting nexin 14 gene. Here we describe a novel SNX14 mutation identified via homozygosity mapping and exome sequencing in a family with characteristics of cerebellar atrophy and intellectual disability.

## 2. Case Report

**Figure 1:**
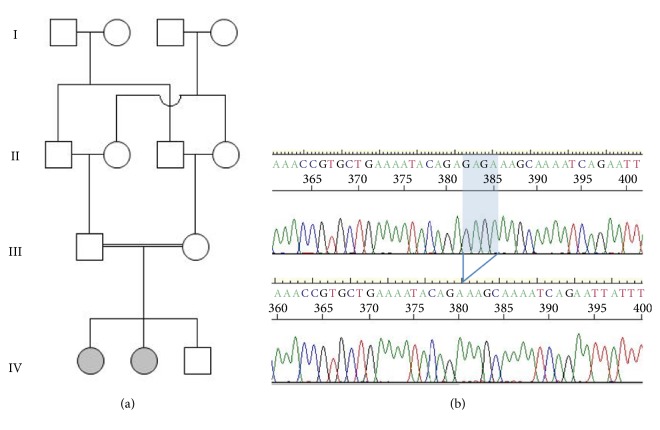
(a) Consanguineous Omani family segregating cerebellar atrophy, ataxia, coarse facial features, and intellectual disability. Arrow indicates index patient (IV-1). (b) Sanger sequencing chromatogram for reference (top panel) and index (bottom) showing the SNX14:c.2319_2322del pathogenic mutation (blue shade). The nature and zygosity status of the c.2319_2322del mutation segregating within this family is confirmed.

**Figure 2:**
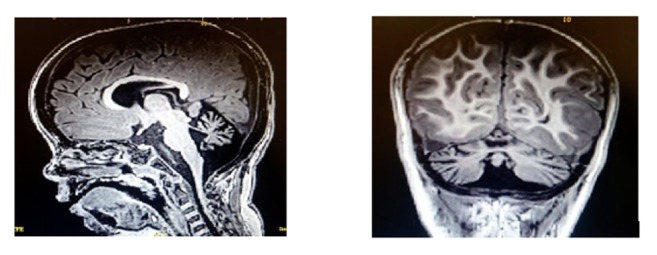
T1-weighed brain MRI of the index patient showing cerebellar atrophy.

This research was approved by Sultan Qaboos University Ethics Committee (SQU-EC/158/14). An Omani family characterized by the presence of cerebellar atrophy and intellectual disability was enrolled for the purpose of identifying the underlying molecular defect [Figure 1(a)]. Our patients are 2 Omani girls born to double first consanguineous parent. The index patient (IV-1), now 6.8 years of age, was born via normal SVD at full term after unremarkable pregnancy. She first came to medical attention for recurrent chest infections since the age of 4 months. Investigations showed bronchomalacia of left main bronchus. Echocardiography showed ASD II, which was closed surgically at 6 months of age. At 20 months of age, global development delay was noticed. She could sit with support but was unable to stand or crawl and could only say a few words. Physical examination showed coarse facial features, depressed nasal bridge, hypertelorism, and epicanthal folds. Neurological examination showed nystagmus, normal muscle bulk, and mild hypotonia. Epileptic seizures were not present. Power and reflexes were however difficult to assess objectively. Laboratory investigations following newborn screen, plasma amino acids, urine organic and amino acids, mucopolysaccharides, and oligosaccharides were all normal. Skeletal survey was unremarkable. Nerve conduction study was normal. MRI brain showed cerebellar atrophy more prominent in anterior vermis with asymmetric white matter volume loss (more in the left side) resulting in crossed cerebellar diaschisis [Figure 2].

The sister of the index patient (IV-2), now 3 years of age, also has hypotonia. Like her sister, physical examination revealed coarse facial features, bitemporal narrowing, and depressed nasal bridge. She had mild hepatosplenomegaly, mild hypotonia, and hyporeflexia; planters were down-going. ENT evaluation revealed sensorineural hearing deficit.

Following homozygosity-guided whole exome sequencing of the index patient, a c.2319_2322del homozygous deletion within the sorting nexin 14 gene (NM_153816.5, GRCh37) emerged as the most likely candidate for the disorder in this family. This frameshift variation in exon 24 of SNX14 is predicted to lead to a premature stop codon and truncated protein p.(Arg774Serfs*∗*10). Sanger sequencing confirmed the nature and homozygous state of the mutation in the proband and her affected sibling [Figure 1(b)]. The obligate carrier status of the parents was also confirmed. One healthy sibling was found not to be homozygote for the mutation. This loss-of-function mutation appears to be novel as not present in ethnically diverse population databases (dbSNP, gnomAD, or ClinVar) nor in a locally maintained database of 100 ethnically matched individuals.

## 3. Discussion

Here we report the first Omani case of a newly characterized and clinically distinguishable form of autosomal recessive cerebellar ataxia due to SNX14 mutation [3, 4]. In this family, a novel c.2319_2322del mutation was identified following exome sequencing. This frameshift variation in exon 24 of SNX14 is predicted to lead to a truncated protein (p.Arg774Serfs*∗*10). The main clinical features were remarkably similar to those already reported in patients with biallelic SNX14 inactivation and included cerebellar ataxia, coarse facial features, and intellectual disability [reviewed in [5]]. Consistent with other forms of recessive ataxias and SNX-related ataxia in particular, a complete loss-of-function mutation as well as early-onset presentation was observed in this family [3–5].

SNX14 gene encodes a member of the sorting nexin family and plays a role in maintaining normal neuronal excitability and synaptic transmission. Owing to their phox phosphoinositide binding domain, members of this family are involved in intracellular trafficking. SNX14 appears to bind phosphatidylinositol (3,5)-bisphosphate, a key component of late endosomes/lysosomes. SNX14 has been proposed to play a role for mediating the fusion of lysosomes with autophagosomes [4]. The autophagosome pathway is used for the cytoplasmic recycling of macromolecules following lysosomal degradation [6]. Biallelic inactivation of SNX14, as well as the recently described defect of SQSTM1 (sequestosome 1) [7], a multidomain scaffolding protein which functions as a selective autophagy receptor, effectively highlights the essential role of the autophagy pathway in preserving normal neuronal function [8]. More recently, a role for SNX14 in neutral lipid homeostasis between the ER, lysosomes, and lipid droplets has also been suggested. This is important as it may provide a pharmacological target for clinical intervention in SCAR20 [9].

## 4. Conclusion

We have described the first occurrence of SNX14 mutation in Oman and add to the list of gene defect leading to syndromic ataxia in this population, which also includes mutations in SACS gene causative of autosomal recessive spastic ataxia Charlevoix-Saguenay type (unpublished). As molecular testing for the pathogenic variants in these genes is clinically and locally available, targeted molecular analysis should be considered in patients meeting diagnostic criteria prior to NGS-based panel and/or exome sequencing.

## Data Availability

(1) dbSNP, The Single Nucleotide Polymorphism Database, is available at www.ncbi.nlm.nih.gov/snp. (2) gnomAD, Genome Aggregation Database, is available at gnomad.broadinstitute.org. (3) ClinVar, Genomic Variation and Its Relationship to Human Health Database, is available at www.ncbi.nlm.nih.gov/clinvar.
